# Screening Auxin Response, In Vitro Culture Aptitude and Susceptibility to *Agrobacterium*-Mediated Transformation of Italian Commercial Durum Wheat Varieties

**DOI:** 10.3390/molecules21111440

**Published:** 2016-10-28

**Authors:** Wilma Sabetta, Cristina Crosatti, Alexandra Soltész, Valentina Di Rienzo, Cinzia Montemurro

**Affiliations:** 1 Sinagri S.r.l., Spin-off, University of Bari, Via Amendola 165/A, Bari 70126, Italy; valentina.dirienzo@gmail.com (V.D.R.); cinzia.montemurro@uniba.it (C.M.); 2 Council for Agricultural Research and Economics, Genomics Research Centre, Via S.Protaso 302, Fiorenzuola d’Arda (PC) 29017, Italy; cristina.crosatti@crea.gov.it; 3 Agricultural Institute, Centre for Agricultural Research, Hungarian Academy of Sciences, Brunszvik u. 2., Martonvásár H-2462, Hungary; soltesz.alexandra@agrar.mta.hu; 4 Department of Soil, Plant and Food Sciences, University of Bari, Via Amendola 165/A, Bari 70126, Italy

**Keywords:** durum wheat, *Agrobacterium*, Dicamba, 2,4-D, pre-culture, immature embryos

## Abstract

The development of a robust *Agrobacterium*-mediated transformation protocol for a recalcitrant species like durum wheat requires the identification and optimization of factors affecting T-DNA delivery and plant regeneration. The purpose of this research was to compare the behavior of diverse durum wheat genotypes during in vitro culture and *Agrobacterium tumefaciens*-mediated transformation, using immature embryos as explants. Apart from plant genotype, two of the main influencing factors for a successful genetic transformation have been examined here, i.e., auxin source (Dicamba and 2,4-D) and duration of the pre-culture period (one, seven and 21 days). The addition of Dicamba to the media in combination with seven days pre-cultivation resulted in a general enhancement of T-DNA delivery for most of the analyzed cultivars, as revealed by β-glucuronidase (GUS) histochemical assay. Although all genotypes were able to produce calli, significant differences were detected in regeneration and transformation efficiencies, since only two (Karalis and Neolatino) out of 14 cultivars produced fertile transgenic plants. The estimated transformation efficiencies were 6.25% and 1.66% for Karalis and Neolatino, respectively, and χ^2^ analysis revealed the stable integration and segregation of the *gus* transgene in T_1_ and T_2_ progenies. This research has demonstrated that, among the influencing factors, genotype and auxin type play the most important role in the success of durum wheat transformation.

## 1. Introduction

Wheat is certainly one of the most important and widespread crops in the world. In particular, tetraploid durum wheat (*Triticum turgidum* L. var. *durum*) is mainly used to produce pasta and semolina for human consumption, while soft wheat (*Triticum aestivum*) is more widely cultivated for flour and bread-making. Over the years, wheat improvement has received considerable attention from plant breeders, whose efforts have mainly focused on increasing the yield potential, quality characteristics, resistance to biotic stresses and tolerance to abiotic stresses, depending on the regional requirement of the crop [1,2]. Scientific approaches have their history in conventional breeding techniques, principally based on the processes of crossing, back crossing and selection, which have the related disadvantages of being time-consuming and laborious. Since modern biotechnologies emerged as a potential tool for agricultural research, their application to members of the *Triticum* genus has contributed towards the more rapid development of improved cultivars.

Initial attempts to introduce exogenous DNA into the wheat genome employed protoplasts as explants and chemical treatment or electroporation as the delivery methodology [3,4,5]. However, the problematic manipulation of protoplasts and the difficulties associated with plantlet regeneration quickly led researchers to shift their attention to mature and immature embryos as starting explants, and the biolistic approach seemed to be the most successful choice for wheat transformation. This was how the first durum wheat transgenic and fertile plant, resistant to the broad spectrum herbicide Basta^®^ (Bayer Cropscience Pty Ltd., Hawthorn East, Victoria, Australia), was obtained in 1992 [6,7].

Once efficient protocols of *Agrobacterium*-mediated transformation [8] were developed in rice and maize, this alternative strategy was also applied to the *Triticum* genus. The pioneering study on bread wheat (cultivar Bobwhite) dates back to 1997, when Cheng and colleagues reported the production of a large number of transgenic plants, with stable integration, expression and inheritance of transgenes [9]. Reported as a simple, low-cost and highly efficient alternative to the direct gene delivery method, the *Agrobacterium* system also includes the advantages of a defined insertion of a distinct DNA segment into the recipient genome and the easy segregation of selection markers [10,11,12]. Since several key factors, such as genotype, explant, media composition, length of pre-culture and co-cultivation, selection markers, bacteria strain, and so on, have been reported to affect the efficiency of *Triticum* genus transformation [9,13,14], a robust *Agrobacterium*-mediated system suitable for durum and soft wheat has not been applied to a large set of wheat genotypes, and routine protocols have been mainly adapted for soft wheat and often for specific cultivars. Recently, Ishida et al. [15] noticed that the list of key factors in wheat transformation was similar to those studied in other cereals, but the achievement of optimal combination of these factors could be critical in wheat, thus speculating that this was the reason for slow progress and limited success in this species. Starting from the publication of Ishida et al. [15], Richardson and colleagues [16] recently reported the achievement high transformation efficiencies not only for soft wheat cultivar Fielder but also for additional wheat germplasm, including two Australian durum wheat cultivars. The restricted number of studies on Italian durum wheat germplasm and the complications with the genetic transformation via *Agrobacterium*-mediated co-cultivation have motivated us to examine three of the most important factors influencing the success of durum wheat transformation. First of all, since transformability could be strongly genotype-dependent in wheat and since varietal differences in efficiency of transformation have not yet been extensively studied, we screened the susceptibility of some Italian commercial durum wheat cultivars to *Agrobacterium* infection, using immature embryos as explants. Second, the effectiveness of different pre-culture timing has been proven. In some publications, mostly regarding soft wheat, the addition of a first step called “pre-culture” is demonstrated to be crucial in successful *Agrobacterium*-mediated transformation and transformation efficiency [9,17,18]. However, the length of pre-culture is usually balanced against medium composition to optimize the stage of cellular differentiation, cell cycle and osmotic conditioning [19]. Third, the effect of two auxins on the success of durum wheat genetic transformation was tested. As extensively studied in both dicot and monocot species, the plant growth regulator included in the medium is an important factor to take into account during a genetic transformation experiment. Generally, media supporting active cell division are preferable [11] and Picloram and 2,4-dichlorophenoxyacetic acid (2,4-D) are reported as the most common auxins [18,20,21,22,23,24]. The effect of another synthetic auxin, the 3,6-dichloro-2-methoxybenzoic acid (Dicamba), has been tested on wheat callus induction and regeneration. In previous research on Russian winter and spring wheat cultivars using mature embryos as explants, Fillipov et al. [25] found Dicamba to be more effective than 2,4-D for embryogenic callus induction. Moreover, increasing the time of exposure and the addition of other auxins significantly strengthened the Dicamba action in terms of embryogenic callus frequency.

The objectives of our work were therefore to investigate the aptitude of some of the most common Italian durum wheat cultivar to *Agrobacterium* infection and transformation and to develop an experimental procedure suitable for immature embryos by the establishment of optimal conditions for two of the key parameters influencing gene transfer. For these purposes, one of the most used visual expression marker gene, i.e., the β-glucuronidase (*gus*) gene [26], has been chosen as a detection marker.

## 2. Results

### 2.1. Callus Induction Rate

**Figure 1:**
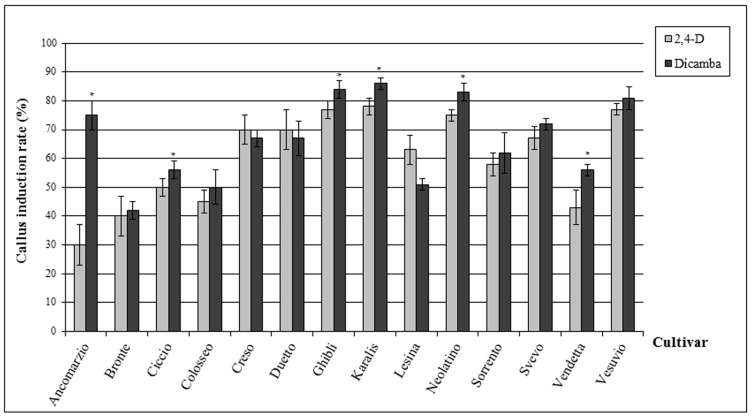
Effect of 2,4-D and Dicamba on callus induction rate (%) in the 14 tested durum wheat cultivars on the 21st day of pre-culture. The data are the means ± standard errors of three independent experiments, analysed by one-way ANOVA test. Asterisks indicate significant differences (*p* ≤ 0.05).

To examine the influence of auxin type first on callus induction, and then on embryogenesis and shoot production capacity of several Italian durum wheat cultivars, two auxins were tested: 2,4-D and Dicamba. A total of 3360 immature embryos were dissected and the callus induction rate was calculated at the end of the pre-culture period, i.e., at the 21st day. It was observed that all the genotypes were able to produce calli, but the degree of callus induction varied (Figure 1). In general, the response of durum wheat varieties was similar to the two auxins, since the callus induction rate ranged from 42% to 86% for Dicamba and from 30% to 78% for 2,4-D. The only exception to this general trend was for cv Ancomarzio, whose callus induction rate was strongly increased by Dicamba (75%) in comparison with 2,4-D (30%). Callus induction due to added auxins was increased the most in cultivars Ghibli, Karalis, Neolatino and Vesuvio, Dicamba being slightly more effective than 2,4-D. Callus induction in the remaining cultivars was also increased by auxins but to a lesser extent.

### 2.2. GUS Assay

**Figure 2:**
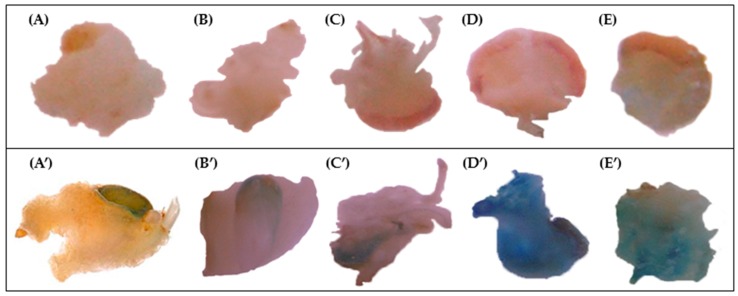
GUS histochemical assay in some of the analysed wheat varieties. Examples of transient *gus* gene expression evaluated in relation to genotype and pre-culturing time, on Dicamba supplemented medium: transformed embryos (letters with apex) were compared with the corresponding untransformed ones (letters without apex). (**A**,**A’**) cv Neolatino after one day; (**B**,**B’**) cv Svevo, (**C**,**C’**) Sorrento and (**D**,**D’**) Ghibli after seven days; and (**E**,**E’**) cv Ghibli after twenty-one days.

The in vitro explant pre-culture has been reported as one of the influencing factors for high efficiency of Agrobacterium-mediated transformation experiments in wheat and several other crops [9,13,14,18,27]. This period is the time between the isolation of the explants and their inoculation with the Agrobacterium culture. Here, the influence of three pre-cultivation periods has been evaluated for each analyzed cultivar. Calli derived from immature embryos were co-cultivated with the *A. tumefaciens* AGL1 hypervirulent strain after one, seven and 21 days of pre-culture. Ten calli per cultivar and per treatment (auxin and pre-culturing time) were sacrificed for the histochemical GUS assay. GUS expression was observed in calli in 13 out of 14 analyzed varieties, and it was found to be strongly genotype-, auxin- and pre-culturing time-dependent (Table 1) (Figure 2). Cultivar Bronte was the most recalcitrant genotype, since no GUS activity was observed in any calli. In general, the shortest (one day) or longest (21 days) pre-culture treatments negatively affected the appearance of blue spots on the analyzed calli. On the contrary, seven days of pre-cultivation was shown to be the most effective treatment, since a large number of small and intense blue stains emerged on the surface of explants of most cultivars, with clear differences in the intensity of colored spots in relation to the auxin treatments. In particular, the highest GUS activity was recorded for cultivars Ghibli and Vesuvio, when grown on both auxin sources, while cultivars Ancomarzio, Creso, Duetto, Neolatino and Sorrento showed the higher blue staining when pre-cultivated on Dicamba added medium.

### 2.3. Plant Regeneration

**Figure 3:**
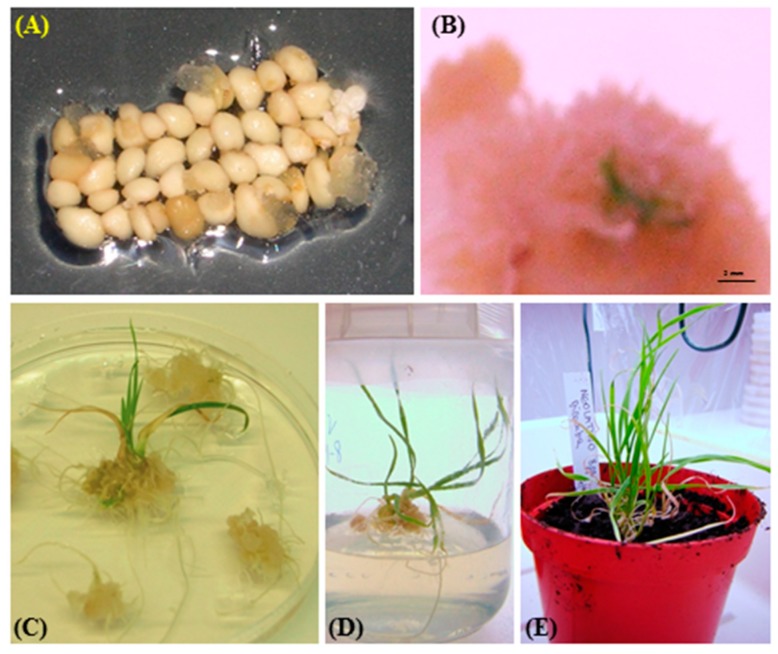
Callogenesis and regenerated plants of Karalis wheat cultivar. (**A**) Callogenesis from pre-cultured embryos; (**B**) embryogenic callus; (**C**) shoots and roots development; (**D**) roots elongation; and (**E**) regenerated plant shifted to soil.

After three days of co-cultivation, the infected calli were subjected to selection in antibiotic-containing medium. During this period, the bacteria were removed and the successfully transformed calli continued to grow vigorously, whereas the untransformed ones started to become necrotic within three weeks. The presence of 6-benzylaminopurine (BAP) in regeneration medium promoted the development of shoot primordia. The selective pressure exerted by the use of antibiotics for the first two regeneration cycles reduced the number of escapes. After a few weeks of sub-cultivation, the nodular structures started to develop into adventitious shoots and clearly emerged from calli (Figure 3). In general, the regeneration ability of immature embryos was rather low among the analyzed wheat cultivars, since only four (Bronte, Karalis, Neolatino and Vesuvio) out of 14 were able to differentiate shoots and roots and mostly from those calli, which originated from seven-day pre-cultured embryos on media containing Dicamba. Surprisingly, cultivar Bronte, i.e., that showed the lowest response to auxin treatments in terms of both callus induction rate and GUS histochemical assay, was among the regenerating cultivars. Generally, the regeneration efficiency ranged between 0% and 21.1%, as listed in Table 2. Although the two tested auxins did not strongly affect the callus induction rate for all the analyzed cultivars, they had a clear effect on regeneration efficiency, concurring with Barro et al. [21] and He and Lazzeri [28]. Indeed, the presence of Dicamba in the substrate generally led to a better response of explants to regeneration medium, especially for cultivars Bronte and Karalis, which were able to produce shoots only on Dicamba supplemented medium. Moreover, the cultivar Karalis showed the highest regeneration percentage (21.1%) followed by cultivar Neolatino (17.8%). Cultivar Vesuvio moderately differed from the other two cultivars in the number of regenerated plants, which resulted as very low on both auxin sources. Successively, all the initiated shoots were matured on half strength and free hormone substrate for root development.

### 2.4. Characterization of Transgenic Wheat Lines

**Figure 4:**
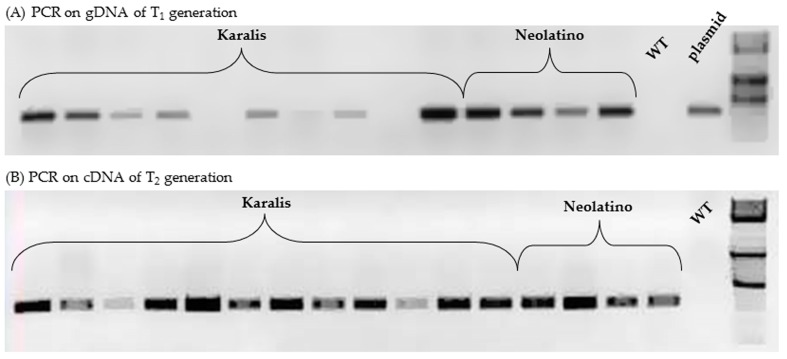
PCR analysis of some progeny of transgenic plants. The presence of the *gus* gene was proven by electrophoresis of amplified product (300 bp) on gDNA from plants of T_1_ families (**A**) and on cDNA from plants of T_2_ progeny (**B**). DNA from non-transformed (control) plants was used as a negative control, while the plasmid (pWBVec10a) carried by the *A. tumefaciens* strain, was used as a positive control. The 1-kb DNA Molecular Weight Ladder is represented in the last lane.

The T_0_ regenerated plants of Bronte, Karalis, Neolatino and Vesuvio cultivars were grown in a growth chamber and evaluated for morphology and fertility. Only lines able to produce spikes were subjected to further studies. Four out of 19 Karalis plants and two out of 18 Neolatino plants were fertile or partially fertile and produced T_1_ seeds, while none of the five Bronte plants and 11 Vesuvio plants were able to reach complete maturity. To confirm the stable integration of T-DNA into the durum wheat genome, several T_1_ plants from different spikes of T_0_ plants were screened by PCR amplification using *gus* gene specific primers. As shown in Figure 4A, some of the analysed plants showed a clear band corresponding to the expected size (300 bp) of the *gus* gene. Plants not showing any PCR product for this gene were considered samples that had escaped selection. Results of PCR screens were subsequently corroborated by PCR product sequencing, thus indicating that the transgene was successfully introduced into Karalis and Neolatino genotypes. The transformation efficiency was calculated as number of T_1_ transformants confirmed by PCR and sequencing analysis of the total number of isolated embryos. In Karalis and Neolatino genotypes, transformation efficiencies were 6.25% and 1.66%, respectively. A simple Mendelian 3:1 ratio for presence/absence of *gus*-amplified band was observed in Neolatino progeny, thus suggesting a single integration site. In the case of Karalis cultivar, 53% of T_1_ plants had a non-Mendelian segregation pattern (1:1 ratio), probably as a consequence of the insertion of two or three linked copies of *gus* gene into the plant genome. Statistical analysis of the progeny also confirmed inheritance and segregation of the transgene at 0.05% significance level. Since the GUS assay on T_2_ leaves indicated no clear β-glucuronidase activity, transgene expression was proven in T_2_ progeny derived from some of the *gus*-positive T_1_ lines for both wheat genotypes, by the amplification of cDNA and PCR-product sequencing (Figure 4B and Table 3).

## 3. Discussion

Here, we report a screening of some of the most common Italian durum wheat cultivars (Ancomarzio, Bronte, Ciccio, Colosseo, Creso, Duetto, Ghibli, Karalis, Lesina, Neolatino, Sorrento, Svevo, Vendetta and Vesuvio) for their ability to undergo *Agrobacterium*-mediated transformation, with particular emphasis on two of the reported influencing factors, i.e., auxin source and duration of the pre-culture period.

It is known that auxins play an important role in the activation of genes involved in cell de-differentiation and division [21,29] and that cells in the S-phase (DNA synthesis phase) of the cell cycle are more predisposed to the integration of foreign DNA [30]. In our study, two types of synthetic auxin were tested: Dicamba and 2,4-D, which have distinct effects on the induction of cell division, proliferation and further regeneration, accordingly to exposure time and concentration [20,21,31]. Generally, both auxins seemed to equally influence the formation of calli, since each wheat genotype responded in a comparable way to both treatments; the only exception was shown by cv Ancomarzio, whose callus formation was only extremely promoted by Dicamba. Moreover, Dicamba-induced calli developed in a shorter time, probably due to Dicamba’s rapid metabolism into the cells of cultured tissues [32]. The frequency of callus induction in the analysed cultivars varied from 30% to 86%, in accordance with what has been observed for soft wheat in several experiments, but was generally higher than that reported for durum wheat [14,33,34,35,36]. Cultivars Ghibli, Karalis, Neolatino and Vesuvio responded well to both auxin stimuli, while cultivars Bronte, Ciccio, Colosseo and Vendetta responded less well to either auxin. Our results suggest that the variable response of cultivars to in vitro culture and auxin treatments could be mainly due to genotype.

To clarify these results, the pedigrees of the considered cultivars have been investigated, except for cv Karalis and Sorrento, for which no information were available (see Supplementary Materials Table S1). As expected, most of the genotypes do not share common ancestors, despite showing similar agro-technological traits. The appearance and growth rate of calli were different among varieties, as an indication of genotypic rearrangements and differences, thus confirming that callus induction mainly depends upon wheat genotype, as also postulated by other authors [37,38,39,40,41,42,43,44,45,46].

In several experiments, a pre-culture step of explants has been tested to clarify its influence on T-DNA delivery into plant tissues, and thereby its effect on their competence for DNA uptake, gene expression and regeneration. The length of the pre-culture period reported by other authors varies according to the explant type and the experimental protocol [47,48,49,50]. With regard to immature embryos of wheat cultivars, the pre-culture time is summarily reported to range from one hour up to 25 days [13,14,18]. Significant differences in transformation efficiencies were found between pre-cultured versus freshly isolated immature embryos [9] and between 14-day versus five-day pre-cultivated embryos [18]. Inflorescences of soft wheat cv Baldus were kept in pre-culture for a long time, up to 39 days, and analysed weekly for GUS activity: the optimal T-DNA delivery was obtained from inflorescences pre-cultured for 21 days [13]. In our study, the effect of three different pre-culture periods prior to inoculation, i.e., one, seven and 21 days, was analysed on the efficiency of durum wheat transformation. Generally, the one-day-old pre-cultured embryos formed few calli, independently of the auxin source; this is in contrast with observations in barley, for which one day pre-culture was enough for embryo adaptation [51]. Additionally, with an extended pre-culture period (21 days), the risk of callus necrosis and microbial contamination obviously increased. The effect of seven-day pre-cultivation before *Agrobacterium* co-cultivation resulted in a general enhancement of T-DNA delivery for most cultivars, in particular in combination with Dicamba-containing media. Probably, the susceptibility of durum wheat embryos to *Agrobacterium* infection was somehow improved by a moderate period of in vitro embryo adaptation. High levels of GUS expression were often found in cultivars with the highest callus induction rate (Ghibli, Neolatino, Vesuvio) and in cultivars Ancomarzio, Creso and Duetto. On the other hand, cv Bronte and Vendetta showed the most recalcitrant genotypes to transient genetic transformation among the tested cultivars.

The 14 durum wheat cultivars, analysed here, differed remarkably in terms of callus induction rate, regeneration efficiency and transformation efficiency, indicating that these variables are not directly linked, as also observed by other authors [24,33,36,52,53]. Surprisingly, those cultivars with medium-high callus induction rate and *gus* gene expression, which were cultivated in a Dicamba containing medium, were unable to regenerate plants. The loss of regeneration potential over time has been reported as a major limitation factor in most wheat-transformation experiments. In our study, only four cultivars (Bronte, Karalis, Neolatino and Vesuvio) succeeded in plant regeneration, respectively, with total regeneration percentages equal to 5.6%, 21.1%, 17.8% and 11.1%. Although the two auxins did not strongly differ in affecting callus induction rate, they had a clear effect on plant regeneration as Dicamba was more effective for embryogenic calli induction and plant regeneration frequency than 2,4-D (mean 13.9% versus mean 0.8%), in agreement with previous studies [21,25,28,32,54,55]. Two (Karalis and Neolatino) out of four regenerated cultivars resulted in fertile plants, allowing us to calculate their transformation efficiencies as 6.25% and 1.66%, respectively (average of 3.9%). This is in agreement with most publications since 2000, reporting transformation frequencies of less than 5% for the *Triticum* genus, which is an order of magnitude lower than that observed in other major cereals [14,56,57,58,59,60,61,62,63]. With specific regard to durum wheat, better results in terms of transformation efficiencies (respectively, 9.7% and 6.3%) were obtained for cultivars Ofanto and Stewart when a super-binary pGreen/pSoup system [64] and increased Picloram and acetosyringone concentrations were applied [65].

## 4. Materials and Methods

### 4.1. Plant Material

Fourteen durum wheat cultivars (Ancomarzio, Bronte, Ciccio, Colosseo, Creso, Duetto, Ghibli, Karalis, Lesina, Neolatino, Sorrento, Svevo, Vendetta and Vesuvio) were sown in a greenhouse with a cultivation distance of 30 and 20 cm inter- and intra-rows respectively, at the “Martucci” experimental field in Valenzano (Bari, Italy). Plants were regularly watered and fertilized. Seven spikes per each cultivar were collected at 12–16 days post-anthesis and stored at 4 °C for 24 h. Immature seeds were first surface-sterilised with 70% (*v*/*v*) ethanol (Sigma Aldrich, Saint Louis, MO, USA) for 2 min, and then with 0.05% (*v*/*v*) HgCl_2_ (Fluka-Sigma Aldrich) and 0.04% Tween 20 (Sigma Aldrich) for 15 min with gentle shaking. The seeds were rinsed with four changes of sterile distilled water for 10 min each.

### 4.2. Embryo Isolation

For each cultivar, 240 immature embryos, about 1.8–2.5 mm long, were isolated in sterile conditions. Forty embryos per plate were pre-cultivated scutellum-side up as described by Wan and Lemaux [66] onto CIM, and kept for 1, 7 and 21 days in darkness at 24 °C. The CIM was prepared with 4.3 g/L ready-to-use salts of Murashige and Skoog medium [67] supplemented with 30 g/L maltose, 1.0 mg/L thiamine-HCl, 0.25 g/L myo-inositol, 1.0 g/L casein hydrolysate, 0.69 g/L proline, 4.9 μM CuSO_4_·5H_2_O, 1 mg/L Picloram, and 3.5 g/L Phytagel. Two different auxin sources were tested: 2.5 mg/L 3,6-dichloro-2-methoxybenzoic acid (Dicamba) and 1 mg/L 2,4-dichlorophenoxyacetic acid (2,4-D). All reagents were supplied by Sigma Aldrich. The callus induction rate was calculated as a percentage ratio between the number of calli and the initial number of isolated embryos per single cultivar and experiment (120).

### 4.3. Agrobacterium tumefaciens Culture

For our experiments, the *Agrobacterium tumefaciens* AGL1 hypervirulent strain [68], carrying the vector pWBVec10a with the Ubi1-P/*gus*/nos3′ cassette, was raised and kindly provided by Dr. MB Wang (CSIRO Plant Industry, Canberra, Australia) as a result of previous collaborations with colleagues from the Council for Agricultural Research and Economics, Genomics Research Centre [69]. In this binary vector system, the pWBVec10a plasmid harboured spectinomycin and hygromycin resistance genes and *gus* gene under the control of maize Ubi1-promoter. Bacteria cultures were grown at 28 °C for 22 h with gently shaking (150 rpm) in 5 mL MG liquid medium (5 g/L mannitol, 1 g/L l-glutamic acid, 0.25 g/L KH_2_PO_4_, 0.10 g/L NaCl, 0.10 g/L MgSO_4_·7H_2_O, 1 ng/mL biotin, 5 g/L tryptone, 2.5 g/L yeast extract, pH 7.0) added with 25 mg/L carbenicillin, 25 mg/L rifampicin and 25 mg/L spectinomycin [70]. When the OD_600_ reached 1.4–1.7, the cultures were used for embryo transformation.

### 4.4. Embryo Transformation and Sub-Cultivation

Three hundred μL of *Agrobacterium* culture were directly spread on the scutella of embryos of each cultivar, pre-cultured on CIM for 1, 7 and 21 days. Co-cultivation was carried out keeping embryos at the centre of the plate for 45 min and then in spatial culture under dark conditions at 24 °C. After 2–3 days of co-cultivation, bacteria were removed by transferring embryos and embryo-derived calli on selective media (CIM added with 50 mg/L Hygromycin and 150 mg/L Timentin (ticarcillin:clavulanic acid (15:1); Smithkline Beecham, Brentford, Middlesex, UK), and by sub-cultivation every two weeks, at 24 °C in darkness.

### 4.5. GUS Activity Assay for Transient Expression

The *gus* gene transient expression was verified by an histo-chemically assay, according to Wu et al. [14]. After 2–3 days of co-cultivation, 10 embryogenic calli per each cultivar were incubated for 2 days at 37 °C in 10 mM Ethylenediaminetetraacetic acid (EDTA) (Sigma Aldrich) buffer containing 1 mM 5-bromo-4-chloro-3-indolyl-glucuronide (X-Gluc) (Clontech Laboratories, Inc.; Mountain View, CA, USA) and then washed with 70% ethanol. The explants with, or without, developed blue spots were recorded (0 = no colour; 1 = light blue; 2 = medium blue; 3 = dark blue).

### 4.6. Plant Regeneration and Rooting

Four regeneration cycles of two weeks each were performed on Wheat Regeneration (WR) medium (2.84 g/L MS without NH_3_NO_3_, 165 mg/L NH_3_NO_3_, 0.4 mg/mL Thiamine-HCl, 100 mg/L Inositol, 730 mg/L Glutamine, 1.223 mg/L CuSO_4_·5H_2_O, 62 g/L Maltose, 3.5 g/L Phytagel, 1 mg/L 6-benzylaminopurine (BAP), pH 5.8) (Dr. H.H. Steinbiss personal communication), using 50 mg/L Hygromycin and 150 mg/L Timentin for the first two cycles. Calli were incubated at 24 °C with 16 h light/8 h darkness until green tissues and/or shoots appeared, and then transferred to fresh rooting media (half-strength CIM, hormone free, no antibiotics). About three weeks later, surviving plantlets were transferred to soil and grown to complete maturity under greenhouse conditions. Ten seeds of each T_1_ spike were cultivated to achieve T_2_ seeds. The regeneration efficiency was calculated as percentage ratio between the number of calli able to regenerate and the number of isolated embryos per single cultivar and experiment (pre-culturing time and auxin source), subtracting the number of sacrificed calli for GUS assay (60 in total).

### 4.7. Characterization of Stable Transgenic Lines

Genomic DNA was isolated from leaf material of untransformed (control) and transformed plants and their progeny using the CTAB (hexadecyltrimethylammonium bromide) method [71]. Amplification reactions were carried out in 20 μL volume containing 50 ng/μL of genomic DNA, 1 X Phusion HF Buffer, 0.2 mM each dNTP, 3% DMSO, 0.5 μM each primer (*gus*-F: 5′-CAATTGCTGTGCCAGGCAGTTT-3′; *gus*-R: 5′-CGGGATAGTCTGCCAGTTCAGTTC-3′) and 0.015 U Phusion High-Fidelity DNA Polymerase (Finnzymes Oy, Vantaa, Finland). The amplification program included an initial denaturation at 98 °C for 30 s, following by 35 cycles at 98 °C for 10 s, 58 °C for 20 s, 72 °C for 30 s, and by a final extension at 72 °C for 10 min. The presence of a 300 bp *gus* fragment was checked by electrophoresing 5 μL of the amplified products on 1% SeaKem^®^ LE Agarose gel (Lonza, Switzerland). Amplicons were purified with DNA purification kit NucleoSpin^®^ PCR Clean-up (Macherey-Nagel, Duren, Germany), quantified by Qubit^®^ Fluorometer (Life Technologies, Carlsbad, CA, USA), and sequenced by Macrogen Europe (Amsterdam, The Netherlands).

Total RNA was extracted from 100 mg of leaf tissue of transgenic plants, which had proven positive PCR results, using the RNeasy Plant Mini kit (Qiagen, Venlo, The Netherlands). The QuantiTect^®^ Reverse Transcription Kit (Qiagen) was used for cDNA synthesis with efficient genomic DNA removal, starting from 1 µg RNA. For *gus* gene expression analysis, the same amplification conditions, which were used for genomic DNA, were applied to cDNA. Transformation efficiency was calculated as percentage ratio between the number of *gus*-PCR-positive regenerated plants and the initial number of isolated embryos per single cultivar and experiment (120).

### 4.8. Transgene Inheritance

Transmission of the transgene to progenies was confirmed using PCR. Segregation ratios were calculated from these data. The *gus* gene inheritance and the number of loci were analysed by an χ^2^ Test, in which the observed values were compared to theoretical values corresponding to the integration of one or more copies of the transgene [72].

## 5. Conclusions

Despite its importance, progress in wheat transformation has lagged behind other major cereal crops, due partially to its recalcitrance to in vitro regeneration and transformation. Many studies seemed to show high levels of experiment-to-experiment variation in efficiency, which is currently hard to control due to the high number of factors influencing stable transformation, including auxin treatment and pre-culture duration. The results of our research imply that durum wheat agronomically important genotypes across the Italian market can induce callus, but not all of them are able to regenerate transgenic plants from immature embryos at the same frequencies reported in literature for standard wheat cultivars used in bioengineering. Both types of auxins used here have influenced the in vitro responses, although Dicamba clearly promoted the success of regeneration of transgenic plants. Similarly, based on transformation results, the tested genotypes could be classified into well-adapted, moderately-adapted or non-adapted cultivars to *Agrobacterium*-mediated transformation, thereby implicating, or not, their potential use for modern genetic breeding.

## Figures and Tables

**Table 1:** Effect of pre-culture duration and auxin treatment on transient *gus* gene expression. The intensity of blue spot staining was evaluated on sacrificed immature embryos as follows: 0 = no colour; 1 = light blue; 2 = medium blue; and 3 = dark blue. Results were the average of the number of blue spots per embryo, considering 10 embryos per cultivar and per treatment (totally 60 embryos per cv).

GUS Spot Staining Intensity						
Cultivar	Dicamba			2,4-D		
	Days of Pre-Cultivation			Days of Pre-Cultivation		
	1	7	21	1	7	21
Ancomarzio	0	3	1	0	1	0
Bronte	0	0	0	0	0	0
Ciccio	1	2	0	1	2	0
Colosseo	0	0	0	0	2	0
Creso	0	3	0	1	2	0
Duetto	0	3	0	0	2	0
Ghibli	0	3	1	0	3	0
Karalis	0	1	0	1	2	0
Lesina	0	2	0	0	2	0
Neolatino	1	3	0	1	1	0
Sorrento	0	2	0	0	1	1
Svevo	0	1	0	0	1	0
Vendetta	0	0	0	0	1	0
Vesuvio	0	3	0	0	3	0

**Table 2:** Cultivar, auxin source and regeneration efficiency. Four out of fourteen durum wheat cultivars were able to regenerate plants; the regeneration percentage per single auxin treatment was calculated as number of T_0_ in vitro obtained plants on 90 originally excised embryos (30 were subtracted because of the GUS activity assay).

Cultivar	Auxin Ource	Regeneration Efficiency (%)
Ronte	2,4-D	0.0
	Dicamba	5.6
Karalis	2,4-D	0.0
	Dicamba	21.1
Neolatino	2,4-D	2.2
	Dicamba	17.8
Vesuvio	2,4-D	1.1
	Dicamba	11.1

**Table 3:** Segregation ratios of the *gus* gene in progenies of T_0_ transformed wheat.

Cultivar	No. T_1_ Plants	No. T_1_ Plants *gus* + by PCR	No. T_1_ Plants *gus* − by PCR	χ^2^ Test (*p* < 0.05)	Segregation Ratio	No. T_2_ Plants *gus* + by PCR on cDNA
Karalis	28	15	13	0.14	1:1	7/15
Neolatio	5	4	1	0.21	3:1	3/4

## Supplementary Materials

Supplementary materials can be accessed at: http://www.mdpi.com/1420-3049/21/11/1440/s1.
