# Supplementary material for: Screening Auxin Response, In Vitro Culture Aptitude and Susceptibility to Agrobacterium-Mediated Transformation of Italian Commercial Durum Wheat Varieties
Source: Molecules. 2016 Oct 28;21(11):1440. doi: 10.3390/molecules21111440 (PMC6274527; doi:10.3390/molecules21111440)
Supplement: Supplementary file 1 [file molecules-21-01440-s001.pdf]

# Supplementary Materials: Screening Auxin Response, In Vitro Culture Aptitude and Susceptibility to *Agrobacterium*-Mediated Transformation of Italian Commercial Durum Wheat Varieties

**Wilma Sabetta, Cristina Crosatti, Alexandra Soltész, Valentina Di Rienzo and Cinzia Montemurro**

**Table S1.** The pedigrees of the 14 analysed durum wheat cultivars are reported.

| Cultivar | Pedigree |
|---|---|
| Ancomarzio | Stotka//Altar84/Ald |
| Bronte | Berillo/Latino |
| Ciccio | F6 Appulo/Valnova//F5 Valforte/Patrizio |
| Colosseo | Mutant Mexa/Creso |
| Creso | Yaktana-54/Norin 10-B//2*Cappelli-63/3/3*Tehuacan-60/4/Capelli-B144 |
| Duetto | 1485/83.74 |
| Ghibli | (Ofanto/Tavoliere)//Primadur |
| Karalis | |
| Lesina | [(Capeiti8/Creso)//Creso]/(Trinakria/Valforte) |
| Neolatino | Latino/Trinakria//MG1433/4/Latino |
| Sorrento | |
| Svevo | CIMMYT's selection/Zenit |
| Vendetta | Creso/Ofanto |
| Vesuvio | Ofanto/Simeto |
